# Activation of Haem-Oxidized Soluble Guanylyl Cyclase with BAY 60-2770 in Human Platelets Lead to Overstimulation of the Cyclic GMP Signaling Pathway

**DOI:** 10.1371/journal.pone.0047223

**Published:** 2012-11-08

**Authors:** Camila B. Mendes-Silverio, Luiz O. S. Leiria, Rafael P. Morganti, Gabriel F. Anhê, Sisi Marcondes, Fabíola Z. Mónica, Gilberto De Nucci, Edson Antunes

**Affiliations:** Department of Pharmacology, Faculty of Medical Sciences, University of Campinas (UNICAMP), São Paulo, Brazil; Maastricht University, The Netherlands

## Abstract

**Background and Aims:**

Nitric oxide-independent soluble guanylyl cyclase (sGC) activators reactivate the haem-oxidized enzyme in vascular diseases. This study was undertaken to investigate the anti-platelet mechanisms of the haem-independent sGC activator BAY 60-2770 in human washed platelets. The hypothesis that sGC oxidation potentiates the anti-platelet activities of BAY 60-2770 has been tested.

**Methods:**

Human washed platelet aggregation and adhesion assays, as well as flow cytometry for *α_IIb_β_3_* integrin activation and Western blot for α1 and β1 sGC subunits were performed. Intracellular calcium levels were monitored in platelets loaded with a fluorogenic calcium-binding dye (FluoForte).

**Results:**

BAY 60-2770 (0.001–10 µM) produced significant inhibition of collagen (2 µg/ml)- and thrombin (0.1 U/ml)-induced platelet aggregation that was markedly potentiated by the sGC inhibitor ODQ (10 µM). In fibrinogen-coated plates, BAY 60-2770 significantly inhibited platelet adhesion, an effect potentiated by ODQ. BAY 60-2770 increased the cGMP levels and reduced the intracellular Ca^2+^ levels, both of which were potentiated by ODQ. The cell-permeable cGMP analogue 8-Br-cGMP (100 µM) inhibited platelet aggregation and Ca^2+^ levels in an ODQ-insensitive manner. The cAMP levels remained unchanged by BAY 60-2770. Collagen- and thrombin-induced *α_IIb_β_3_* activation was markedly inhibited by BAY 60-2770 that was further inhibited by ODQ. The effects of sodium nitroprusside (3 µM) were all prevented by ODQ. Incubation with ODQ (10 µM) significantly reduced the protein levels of α1 and β1 sGC subunits, which were prevented by BAY 60-2770.

**Conclusion:**

The inhibitory effects of BAY 60-2770 on aggregation, adhesion, intracellular Ca^2+^ levels and *α_IIb_β_3_* activation are all potentiated in haem-oxidizing conditions. BAY 60-2770 prevents ODQ-induced decrease in sGC protein levels. BAY 60-2770 could be of therapeutic interest in cardiovascular diseases associated with thrombotic complications.

## Introduction

Platelets are a specialized set of blood cells that are an integral component of hemostasis and thrombosis. Platelets are activated by adhesive proteins and soluble agonists through receptor-specific platelet activation pathways inducing the “inside-out” signaling process that lead to activation of the ligand binding function of integrin *α_IIb_β_3_* [1], [2]. Ligand binding to this integrin mediates platelet adhesion and aggregation, and triggers the “outside-in” signaling, resulting in platelet biochemical and morphological responses linked to cytoskeletal remodeling [1], [2]. Endothelial cell-derived nitric oxide (NO) exerts an inhibitory effect in the platelet function by activation of soluble guanylyl cyclase (sGC) –3,5-cyclic guanosine monophosphate (cGMP) signaling pathway, thus preventing adhesion and aggregation of platelets to the vascular wall [3], [4].

Soluble guanylyl cyclase is a heterodimeric complex consisting of two subunits, α and β, each of which contains three common domains, namely, the N-terminal haem-binding domain that mediates the NO sensitivity of the enzyme, the dimerization domain that exists in the middle of the structure for each subunit, and the C-terminal catalytic domain, which is the most highly conserved region between the subunits, and is responsible for the conversion of GTP to cGMP [5]. The α_1_ and β_1_ subunits of sGC are the best characterized in platelets [6]. Nitric oxide binds to the prosthetic group containing the reduced Fe^2+^ haem moiety in sGC, leading to intracellular accumulation of the second messenger molecule cGMP. Cyclic GMP-dependent protein kinases (cGK Iα/cGK Iβ) activation ultimately reduces cytosolic Ca^2+^ levels, thus inhibiting the platelet function [7], [8]. Removal or oxidation of the haem moiety leads to the NO-insensitive form of the enzyme [9].

Nitric oxide-independent sGC agonists have emerged as valuable tools to elucidate the physiopathology of the NO–cGMP signaling pathway. To date, these agonists comprise the so-called sGC stimulators and sGC activators, the former of which depend on the presence of the reduced haem (Fe^2+^) prosthetic moiety within sGC, whereas the latter induces sGC activation when haem iron is in its oxidized state (Fe^3+^ instead of Fe^2+^) or whose haem group is missing [5], [10], [11], [12], [13]. NO-independent, haem-dependent stimulators of sGC include the compounds BAY 41-2272 and BAY 41-8543 [14], [15]. These compounds strongly synergize with NO, stabilizing the nitrosyl-haem complex to stimulate sGC activity up to 200-fold [16] and produce potent vasodilatory and anti-platelet effects [14], [17], [18]. Generally, oxidation of haem moiety (Fe^3+^) of sGC with ODQ [19] prevents the smooth muscle relaxations and anti-platelet activities induced by endogenous NO, NO donors and BAY 41-2272 [20]–[23].

Activators of sGC (haem-independent compounds) include the compounds BAY 60-2770, BAY 58-2667 and HMR-1766 [11], [24]–[28]. BAY 58–2667 protects sGC from haem oxidation-induced ubiquitination and proteasomal degradation in rat vascular tissues [29]. Moreover, BAY 60-2770 was recently shown to activate sGC selectively oxidized or devoid of the haem group in superior mesenteric artery of haem oxygenase-1 knockout mice [30]. BAY 60-2770 was also shown to decrease the pulmonary and systemic arterial pressures in rats, which is enhanced by treatment with ODQ [31]. Although these sGC activators may be of potential interest for the treatment of cardiovascular diseases associated with platelet dysfunction [32]–[34], little is known about their actions on platelet reactivity. Therefore, in the present study, we have investigated the inhibitory mechanisms of BAY 60-2770 in aggregation, adhesion, cyclic nucleotide production, intracellular Ca^2+^ mobilization and integrin *α_IIb_β_3_* (GPIIb/IIIa) activation in human washed platelets. We have tested the hypothesis that oxidation of the sGC haem with ODQ rather potentiates the anti-platelet activities of BAY 60-2770.

## Results

### Anti-aggregating effects of BAY 60-2770 in human washed platelets

**Figure 1:**
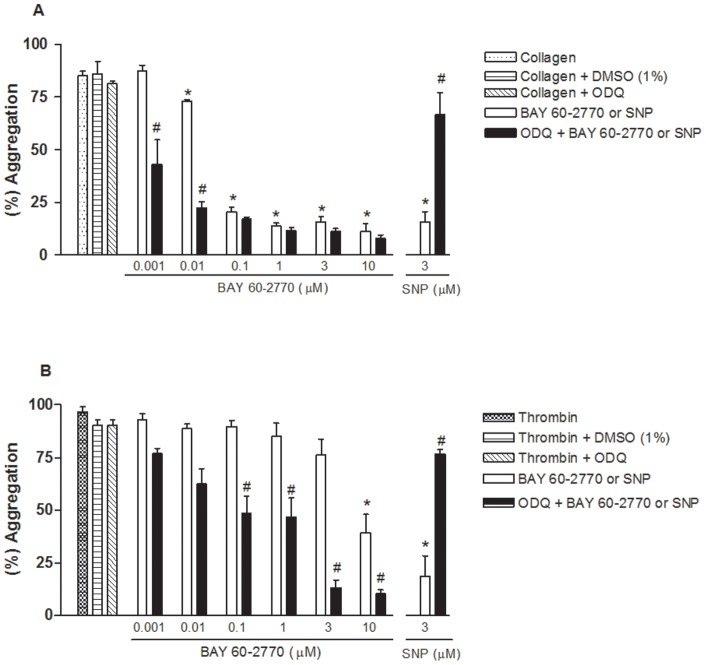
Prior incubation with ODQ potentiates the inhibitory effects of BAY 60-2770 (0.001–10 µM) in human washed platelet aggregation. Platelet suspension (1.2×10^8^ platelets/ml) was pre-incubated for 3 min with the soluble guanylyl cyclase inhibitor ODQ (10 µM) or its correspondent vehicle DMSO (0.5% v/v). Platelets were incubated with BAY 60-2770 (0.001 to 10 µM), DMSO (0.5% v/v) or sodium nitroprusside (SNP, 3 µM) for another 3 min. Platelets were then stimulated with either collagen (2 µg/ml; Panel A) or thrombin (0.1 U/ml; Panel B) to perform the aggregation assays. Note that 1% DMSO (vehicle used for BAY 60-2770 and ODQ) had no effect on collagen- and thrombin-induced platelet aggregation. Data are shown as mean values ± SEM (n = 4–5 individuals). *p<0.05 compared with control values. ^#^p<0.05 compared with respective values in the absence of ODQ.

Incubation of human washed platelets with BAY 60-2770 (0.001–10 µM) produced a significant inhibition of collagen (2 µg/ml)-induced aggregation (Figure 1A). The platelet aggregation was nearly abolished by BAY 60-2770 at concentration ≥0.1 µM. The inhibitory effects of BAY 60-2770 was significantly enhanced (p<0.05) by prior incubation of platelets with the soluble guanylyl cyclase inhibitor ODQ (10 µM), as assessed at concentrations of 0.001 and 0.01 µM (Figure 1A). Sodium nitroprusside (3 µM) largely reduced the collagen-induced platelet aggregation, an effect restored by prior incubation with ODQ (Figure 1A).

Thrombin (0.1 U/ml)-induced platelet aggregation was resistant to BAY 60-2770 at most of the concentrations employed, except at 10 µM where an inhibition by about of 60% (p<0.05) was detected (Figure 1B). However, in the presence of ODQ (10 µM), BAY 60-2770 produced concentration-dependent inhibition of thrombin-induced aggregation in all concentrations assayed (Figure 1B). Sodium nitroprusside (3 µM) largely reduced the thrombin-induced aggregation, which was restored by ODQ (Figure 1B).

**Figure 2:**
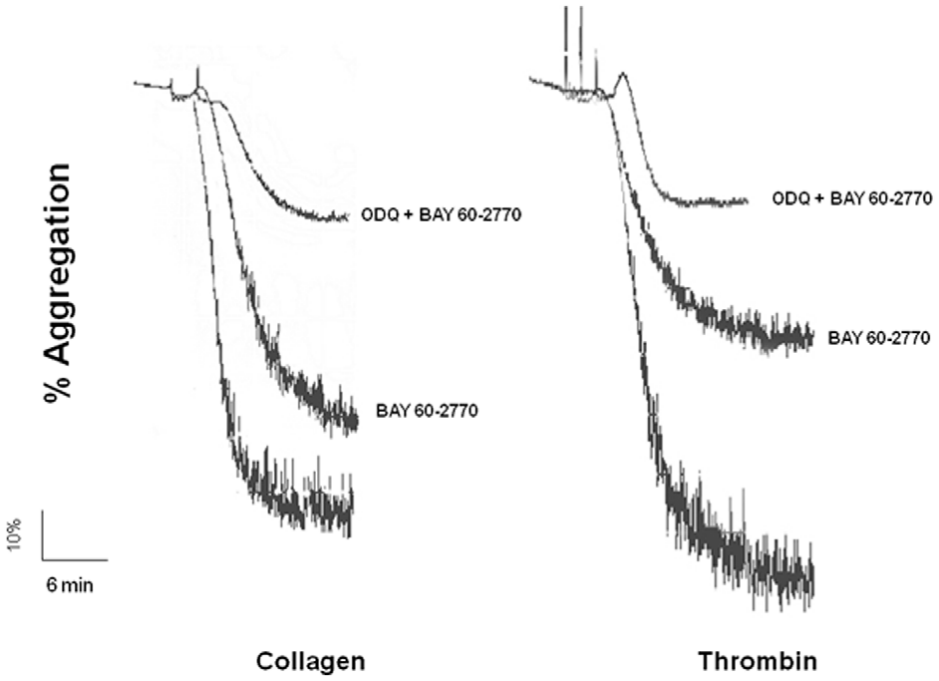
Original tracings showing human washed platelet aggregation stimulated with collagen or thrombin. Platelet suspension (1.2×10^8^ platelets/ml) was pre-incubated with the soluble guanylyl cyclase inhibitor ODQ (10 µM, 3 min). Platelets were then incubated with BAY 60-2770 (0.01 μM for collagen and 10 μM for thrombin), after which they were stimulated with either collagen (2 µg/ml) or thrombin (0.1 U/ml).

Representative tracings of the anti-aggregating effects of BAY 60-2770 in the absence and the presence of ODQ are shown in Figure 2.

The vehicle DMSO alone (1%) affected neither the collagen- nor thrombin-induced platelet aggregation. Incubation with ODQ alone (in the absence of BAY 60-2770 or SNP) also had no effect in collagen- or thrombin-induced platelet aggregation.

In separate experiments, washed platelets were pre-incubated for 3 min with ODQ (10 µM) or DMSO (0.5% v/v). Platelets were then incubated with the cell permeable cGMP analogue 8-Br-cGMP (100 µM) for another 20 min. Platelets were then stimulated with either collagen (2 µg/ml) or thrombin (0.1 U/ml) to perform the aggregation assays (n = 5–7). In the absence of ODQ, 8-Br-cGMP significantly inhibited the collagen- and thrombin-induced aggregation (66±9.2% and 29±10% inhibition, respectively; p<0.05). The inhibition of platelet aggregation by 8-Br-cGMP was not significantly modified by prior incubation with ODQ (65±12% and 35±6.8% inhibition, respectively). The inhibition by 8-Br-cGMP on collagen-induced aggregation was greater compared with thrombin in the presence or absence of ODQ (p<0.02).

### Inhibitory effects of BAY 60-2770 on platelet adhesion to fibrinogen-coated plates

The human platelet adhesion assay is based on the measurement of acid phosphatase activity; therefore, a set of experiments was initially carried out to exclude the possibility that BAY 60-2770 directly affects this enzyme activity. Platelets (1.2×10^8^ platelets/ml; 50 µl per well) were added to uncoated plates and incubated with BAY 60-2770 (0.001 to 10 µM) for 30 min, after which phosphatase activity was measured. BAY 60-2770 did not affect this enzyme activity (data not shown; n = 3), thus validating the adhesion assays in fibrinogen-coated plates, as shown below.

**Figure 3:**
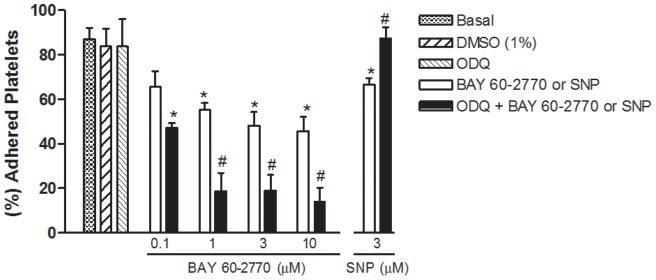
Inhibitory effect of BAY 60-2770 and sodium nitroprusside (SNP) on human platelet adhesion to fibrinogen-coated plates. Platelets (1.2×10^8^ platelets/ml) were pre-incubated or not with ODQ (10 µM, 3 min) and subsequently with BAY 60-2770 (0.1–10 µM) or SNP (3 µM). Platelets were allowed to adhere to the wells for 30 min at 37°C. Note that 1% DMSO (vehicle used for BAY 60-2770 and ODQ) had no effect on platelet adhesion. Data are shown as percent of adhered platelets relative to the maximum adhesion in untreated platelets. Results are shown as mean ± SEM values (n = 4–6 individuals, each performed in triplicate). *p<0.05 compared with control values (in absence of BAY 60-2770 or SNP). #p<0.05 compared with respective values in absence of ODQ.

Figure 3 shows that significant platelet adhesion was observed when cells were kept on fibrinogen-coated plates for 30 min (p<0.05). Incubation with BAY 60-2770 (0.1–10 µM) significantly (p<0.05) inhibited the platelet adhesion, an effect largely potentiated by prior incubation with ODQ (10 µM). Sodium nitroprusside (3 µM) significantly inhibited the platelet adhesion, an effect rather reversed by ODQ (Figure 3).

Incubation of platelets with DMSO (1%) had no effect on platelet adhesion (Figure 3). Incubation with ODQ alone (in the absence of BAY 60-2770 or SNP) also had no effect on platelet adhesion.

### Levels of cGMP and cAMP in human washed platelets

**Figure 4:**
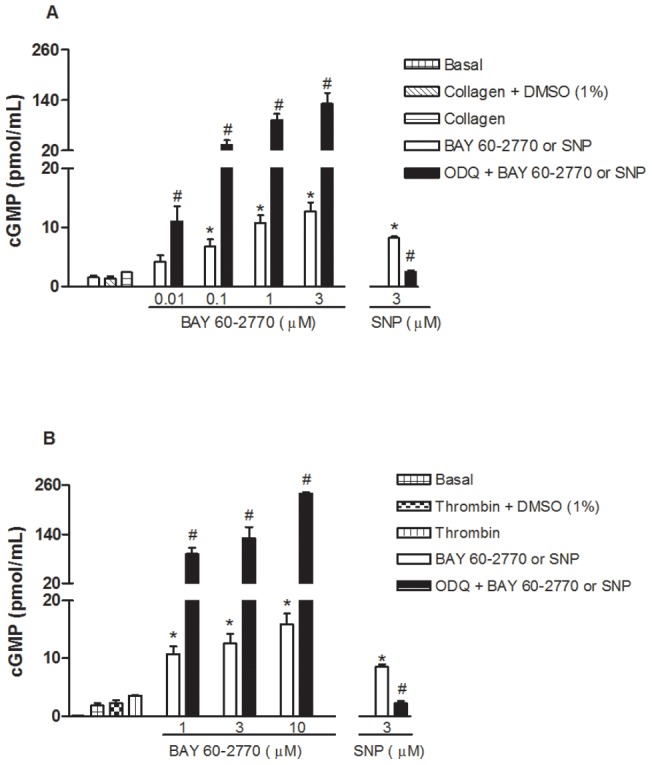
Effect of BAY 60-2770 on cyclic GMP levels in human washed platelets activated with collagen or thrombin. Platelets (1.2×10^8^ platelet/ml) were incubated with BAY 60-2770 (0.01–3 µM for collagen; 1–10 µM for thrombin) or its correspondent vehicle (1% DMSO) in the absence and in the presence of ODQ (10 µM). Platelets were then activated with collagen (2 μg/ml; Panel A) or thrombin (0.1 U/ml; Panel B). Results represent the mean values ± SEM (n = 3 individuals, each performed in triplicate). *p<0.05 compared with control values (in absence of BAY 60-2770); ^#^p<0.05 compared with respective values in absence of ODQ.

The basal cGMP content of human washed platelets (1.8±0.25 pmol/ml) was not significantly affected by thrombin (0.1 U/ml: 2.3±0.5 pmol/ml) or collagen (2 µg/ml: 1.4±0.3 pmol/ml). Incubation with BAY 60-2770 (0.01 to 3 µM) concentration-dependently increased the cGMP levels in collagen-activated platelets (Figure 4A). Similar levels of cGMP by pre-incubation with BAY 60-2770 (1–10 µM) were observed in thrombin-activated platelets (Figure 4B). Prior incubation with ODQ (10 µM) markedly potentiated (p<0.05) the increases in cGMP by BAY 60-2770 in all concentrations tested, as observed in both collagen (Figure 4A) and thrombin (Figure 4B)-activated platelets. Sodium nitroprusside (3 µM) significantly increased the cGMP levels in collagen (Figure 4A) and thrombin-activated platelets (Figure 4B), and pretreatment with ODQ restored the cGMP levels to baseline.

The basal cAMP content of human washed platelets (18±2 pmol/ml) was not significantly affected by stimulation with thrombin or collagen (18±1.4 and 19±2 pmol/ml, respectively). In addition, BAY 60-2770 (10 μM) had no significant effect in the cAMP levels (Table 1). Iloprost (0.3 μM), a stable analogue of prostacyclin (used as a positive control), increased by approximately 23.0-fold the cAMP levels (p<0.01), which was significantly attenuated by prior incubation with the adenylate cyclase inhibitor SQ-22536 (100 µM; Table 1).

**Table 1:** Levels of cyclic AMP in human activated platelets (1.2×10^8^ platelet/ml) treated with BAY 60-2770 or iloprost in the absence and in the presence of the adenylyl cyclase inhibitor SQ-22536 (SQ, 100 μM).

	Cyclic AMP (pmol/ml)
Basal (DMSO 0.5%)	18±2.0
Collagen (2 µg/ml)	19±2.0
Thrombin (0.1 U/ml)	18±1.4
BAY 60-2770 (10 µM)	23.5±1.8
SQ + BAY 60-2770 (10 µM)	24±0.1
Iloprost (0.3 µM)	422±53^*^
SQ + iloprost (0.3 µM)	65±9.3^*#^

Data are shown as mean values ± SEM (*n* = 6). ^*^p<0.01 compared with basal value. ^#^p<0.05 compared with iloprost value.

### Inhibitory effect of BAY 60-2770 in intracellular Ca^2+^ levels [Ca^2+^]_i_

Ca^2+^ mobilization were examined in the human washed platelets activated with either collagen (2 µg/ml) or thrombin (0.1 U/ml) after pre-incubation with BAY 60-2770 or SNP, in the absence and in the presence of ODQ (10 µM).

**Figure 5:**
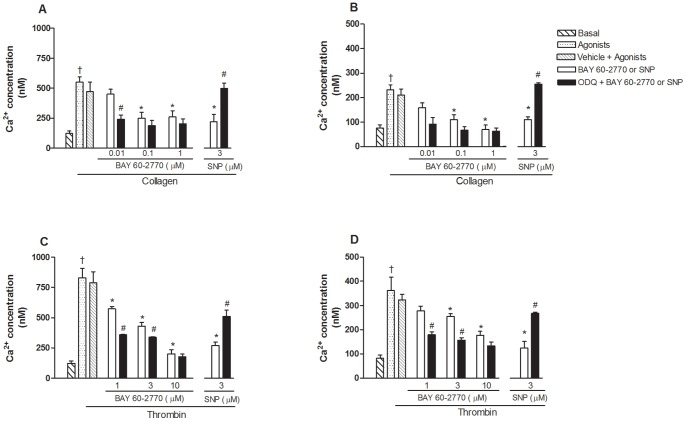
Inhibitory effect of BAY 60-2770 and sodium nitroprusside (SNP) on intracellular Ca^2+^ levels in human washed platelets activated with collagen or thrombin. Platelets (1.2×10^8^ platelets/ml) loaded with Fluorfort (10 µM) were pre-incubated or not with ODQ (10 µM, 3 min) and subsequently with BAY 60-2770 (0.01–10 µM, 3 min) or SNP (3 µM, 3 min) before addition of either collagen (2 μg/ml; Panels A and B) or thrombin (0.1 U/ml; Panels C and D). Assays were carried out in the presence of Ca^2+^ (1 mM) to yield total influx of Ca^2+^ (Panels A and C) or in Ca^2+^-free medium with EGTA to yield Ca^2+^ mobilization from internal storage sites (Panels B and D). Results represent the mean values ± SEM (n = 4–7 individuals). *p<0.05 compared with control values (in absence of BAY 60-2770 or SNP); #p<0.05 compared with respective values in absence of ODQ. ^†^p<0.05 compared with collagen values in panels A and B.

The intraplatelet Ca^2+^ levels were greater (p<0.05) in thrombin-compared with collagen-activated platelets, as detected in both the presence (Figure 5A and C) and absence of Ca^2+^ (Figure 5B and D).

In the presence of 1 mM CaCl_2_, activation of platelets with collagen increased by approximately 6.0-fold the total [Ca^2+^]_i_ (p<0.05; Figure 5A). Incubation with BAY 60-2770 (0.01 to 1 µM) significantly reduced the total [Ca^2+^]_i_, which was further reduced by ODQ, as detected at 0.01 µM (Figure 5A). To verify the intracellular [Ca^2+^]_i_ levels from internal storage sites alone, a Ca^2+^-free Krebs solution in the presence of EGTA (10 µM) was used. Under this condition, the rise in internal Ca^2+^ levels by collagen was still elevated, but to a lower extent when compared to samples in the absence of EGTA (Figure 5B). In addition, BAY 60-2770 concentration-dependently reduced the internal [Ca^2+^]_i_, which was restored to baseline with ODQ (Figure 5B).

Activation of platelets with thrombin also significantly increased the total [Ca^2+^]_i_ (p<0.05; Figure 5C). Incubation with BAY 60-2770 (1 to 10 µM) concentration-dependently reduced the total [Ca^2+^]_i_, that was further reduced by ODQ (Figure 5C). In the presence of EGTA (10 µM), the rise in internal Ca^2+^ levels by thrombin was about of 50% lower compared to samples in the absence of EGTA (Figure 5D). In EGTA-treated platelets, BAY 60-2770 concentration-dependently reduced the internal Ca^2+^ mobilization, an effect potentiated by ODQ (Figure 5D).

Sodium nitroprusside (3 µM) significantly reduced the [Ca^2+^]_i_ in collagen- and thrombin-activated platelets in both the presence (Figure 5A and C) and absence of Ca^2+^ (Figure 5 B and D). Prior incubation with ODQ reversed the reduction of [Ca^2+^]_i_ by SNP.

Incubation of platelets with 1% DMSO had no effect on [Ca^2+^]_i_ in any experimental condition used (Figure 5).

**Figure 6:**
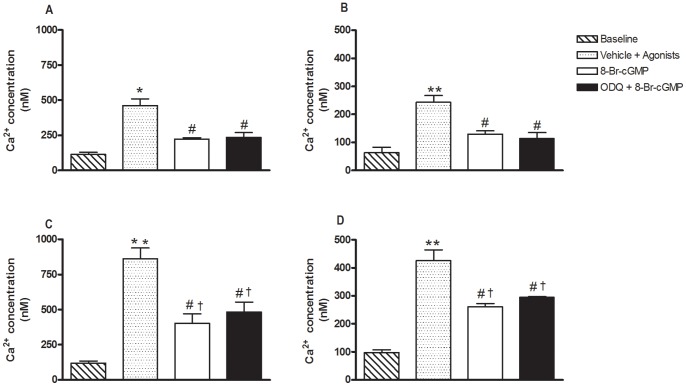
Inhibitory effect of 8-bromo-cyclic GMP on intracellular Ca^2+^ levels in human washed platelets activated with collagen or thrombin. Platelets (1.2×10^8^ platelets/ml) loaded with Fluorfort (10 µM) were pre-incubated or not with ODQ (10 µM, 3 min) and subsequently with 8-Br-cGMP (100 µM, 20 min) before addition of either collagen (2 μg/ml; Panels A and B) or thrombin (0.1 U/ml; Panels C and D). Assays were carried out in the presence of Ca^2+^ (1 mM) to yield total influx of Ca^2+^ (Panels A and C) or in Ca^2+^-free medium with EGTA to yield Ca^+2^ mobilization from internal storage sites (Panels B and D). Results represent the mean values ± SEM (n = 3–5 individuals). *p<0.01, **p<0.001 compared with respective baseline; #p<0.01 compared with respective vehicle. ^†^p<0.01 compared with respective baseline.

In separate experiments, washed platelets were pre-incubated for 3 min with ODQ (10 µM) or DMSO (0.5% v/v). Platelets were then incubated with the cell permeable cGMP analogue 8-Br-cGMP (100 µM) for another 20 min. Platelets were then stimulated with either collagen (2 µg/ml) or thrombin (0.1 U/ml) to perform the Ca^2+^ mobilization assays. In collagen-activated platelets, 8-Br-cGMP nearly restored to baseline the Ca^2+^ levels (either in the absence or the presence of EGTA), and such effects were not modified by prior incubation with ODQ (Figure 6A and B). In thrombin-activated platelets, 8-Br-cGMP significantly inhibited the Ca^2+^ levels either in the absence or the presence of EGTA, but Ca^2+^ levels remained significantly elevated compared with baseline. Pre-incubation with ODQ did not affect the reductions in Ca^2+^ levels by 8-Br-cGMP (Figure 6C and D).

### Integrin *α_IIb_β_3_* (GPIIb/IIIa) activation by PAC-1 analysis

**Figure 7:**
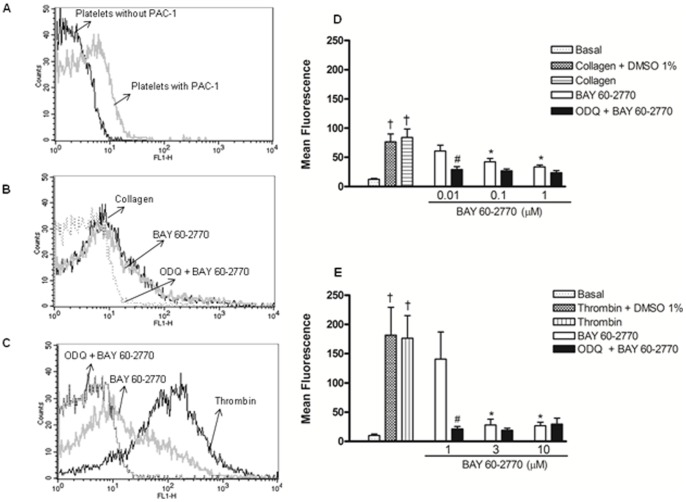
Inhibitory effect of BAY 60-2770 upon PAC-1 mean fluorescence (activated *α_IIb_β_3_*). Platelet suspension (20 µl; 1.2×10^8^ platelets/ml) was pre-incubated with ODQ (10 µM) and BAY 60-2770 or respective vehicle (1% DMSO). Platelets were incubated with PAC-1 solution (FITCPAC-1; 10 µl of 25 µg/ml) or control antibody solution (FITC Mouse IgM, same dilution of PAC-1 solution). Platelets were activated with either collagen (2 μg/ml) or thrombin (0.1 U/ml). Mean fluorescence was acquired in flow cytometer (FACSCalibur) equipped with a 488 nm wavelength argon laser using the FL1 channel. Mean fluorescence was considered as a parameter to describe binding intensity to FITC labeled PAC-1. Panels A, B and C show, respectively, the fluorescence intensity in non-activated, collagen- and thrombin-activated platelets. Panels D and E show the mean fluorescence ± SEM (n = 3–4 individuals). *p<0.05 compared with untreated platelets. ^#^p<0.05 compared with respective values in the absence of ODQ. ^†^p<0.05 compared with respective collagen values in panel D.

Thrombin and collagen induced platelet *α_IIb_β_3_* activation in comparison with non-activated platelets, but thrombin promoted higher *α_IIb_β_3_* activation in comparison with collagen (Figure 7). Pre-treatment with BAY 60-2770 (0.01–1 µM) significantly reduced (p<0.05) the collagen-induced *α_IIb_β_3_* activation, which was further reduced by the presence of ODQ, as evidenced at 0.01 µM of BAY 60-2770 (Figure 7B and D). In thrombin-activated platelets (Figure 7C and E), the inhibitory effects of BAY 60-2770 on *α_IIb_β_3_* activation were also potentiated by ODQ, as evidenced at 1 µM (p<0.05). The control antibody (FITC mouse IgM) ligation was not affected (p<0.05) by thrombin, collagen, BAY 60-2770 or ODQ (data not show; *n* = 3).

### Protein expression of α1 and β1 sGC subunit in washed human platelets

**Figure 8:**
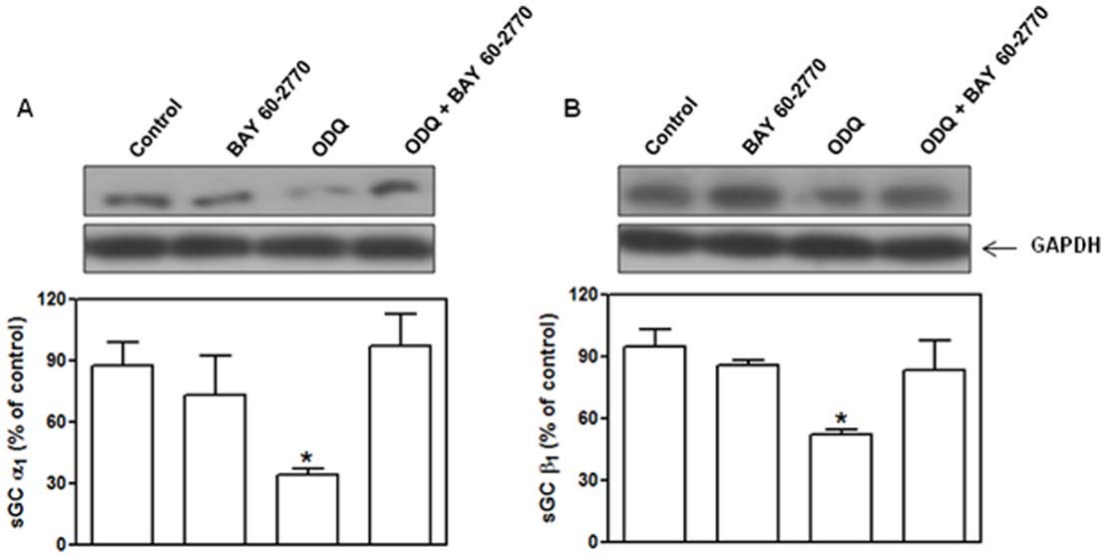
Protective effect of Bay 60-2770 on the ODQ-induced sGC degradation. Isolated platelets were incubated with ODQ (10 µM) in the absence and in the presence of BAY 60-2770 (10 µM) for 2.5 h, after which protein expression of α1 (Panel A) and β1 (Panel B) subunits of sGC subunits were determined by Western blotting. Results represent the mean values ± SEM (n = 4 individuals). *p<0.05 compared with other groups.

Previous studies indicated that oxidation-induced degradation becomes prominent when the incubation time exceeds 2 h [11], [32]. Therefore, washed platelets were incubated with ODQ (10 µM) in the absence and in the presence of BAY 60-2770 (10 µM) for 2.5 h, after which levels of sGC protein for α1 and β1 sGC subunits were determined. Control samples were incubated with DMSO (0.5% v/v). Incubation with ODQ alone reduced by 53% and 40% (p<0.05) the protein levels of α1 and β1 sGC subunits in the human platelets, respectively. Incubation with BAY 60-2770 alone did not affect the protein levels of α1 and β1 sGC subunits; however, BAY 60-2770 fully prevented the reductions by ODQ of the protein levels for both sGC subunits (Figure 8).

## Discussion

This study shows that the haem-independent sGC activator BAY 60-2770 inhibits aggregation, adhesion, intracellular Ca^2+^ levels and *α_IIb_β_3_* activation in human washed platelets activated with collagen and thrombin via cGMP production. Oxidation of the sGC haem moiety with ODQ significantly enhances the inhibitory effects of BAY 60-2770 in all functional and molecular assays tested due to overstimulation of the cGMP signaling pathway. Moreover, ODQ significantly reduces the protein levels of α1 and β1 sGC subunits in human platelets that are prevented by BAY 60-2770.

In our study, BAY 60-2770 was approximately 100 times more potent to inhibit collagen-compared with thrombin-induced platelet aggregation. A maximal inhibition of collagen-induced platelet aggregation by BAY 60-2770 was achieved at 0.1 µM, whereas a 100-times higher concentration (10 µM) was required to produce a significant inhibition of thrombin-induced aggregation. Stimulators of sGC such as BAY 41-2272 and BAY 41-8543 have been shown to be less effective in preventing thrombin-induced platelet aggregation compared with other agents such as ADP and collagen [23]. In our study, under no oxidation with ODQ, similar increases of cGMP levels were observed in platelets stimulated by collagen and thrombin at the same molar concentrations of BAY 60-2770. This excludes the possibility that the different profile of BAY 60-2770 in inhibiting collagen and thrombin-induced responses reflects variations in the levels of this cyclic nucleotide, which is more likely to reflect the mechanisms of platelet activation by one or another platelet agonist. Our findings that the cell permeable cGMP analogue, 8-Br-cGMP, produces a greater inhibition on collagen-compared with thrombin-activated platelets reinforce this suggestion.

Thrombin interacts with PAR-1, PAR-3 or PAR-4 G-protein coupled receptors in the platelet surface [36], whereas collagen activates glycoprotein Ia/IIa (GPIa/IIa or integrin α_2_β_1_) and glycoprotein VI (GPVI) [37]. Following receptor activation, phospholipase C (PLC) cleaves phosphatidylinositol 4,5-bisphosphate (PIP_2_) to the second messengers inositol 1,4,5-trisphosphate (IP_3_) that in turn increases the cytosolic Ca^2+^ concentration by releasing stored Ca^2+^ from the dense tubular system [38]. The amount of Ca^2+^ released from endoplasmic reticular to platelet cytosol is more critical for platelet activation, contributing to various steps of cellular activation, such as reorganization of the actin cytoskeleton necessary for shape change, aggregation, adhesion and conversion of receptor *α_IIb_β_3_* integrin from the low affinity to the high affinity state [39]. We have therefore used a fluorogenic calcium-binding dye assay optimized for cell-permeability and retention (FluoForte) to evaluate the Ca^2+^ mobilization in platelets activated by collagen and thrombin in the presence of BAY 60-2770 and/or ODQ. Both of these agonists raised significantly the Ca^2+^ levels, but thrombin promoted a higher [Ca^2+^]i elevation, even in conditions of absence of extracellular Ca^2+^ (omission of Ca^2+^ in Krebs solution plus addition of EGTA). BAY 60-2770 was more potent to reduce [Ca^2+^]i in collagen-compared with thrombin-activated platelets. Accordingly, lower BAY 60-2770 concentrations (0.01 to 1 µM) were necessary to inhibit Ca^2+^ levels in collagen-compared with thrombin-activated platelets that required higher concentrations of BAY 60-2770 to inhibit Ca^2+^ levels (1 to 10 µM), as detected either in the absence and in the presence of ODQ. Additionally, when analyzing data at 1 µM, BAY 60-2770 almost restored to baseline the Ca^2+^ levels in collagen-activated platelets, but it never reached the baseline in thrombin-activated platelets. The reduction of Ca^2+^ levels by 8-Br-cGMP was also more efficient in collagen-compared with thrombin-activated platelets. The flow cytometry analysis (PAC-1) showed marked integrin α_Ib_β_3_ activation in the presence of either collagen or thrombin, as expected. However, this integrin activation was significantly higher in thrombin-compared with collagen-activated platelets. Again, BAY 60-2770 was more potent to inhibit *α_IIb_β_3_* activation in collagen-activated platelets, which may be attributed to the lower Ca^2+^ levels under activation with this agonist as compared with thrombin.

One essential pre-requisite for the NO-induced activation in sGC is the presence of the reduced haem group [5]. Oxidation of haem moiety (Fe^3+^) with ODQ renders the enzyme insensitive to endogenous NO, thus preventing the vascular and non-vascular smooth muscle relaxations [20]–[22], as well as the anti-platelet effects in response to sGC stimulators, including BAY 41-2272 [23]. In contrast to BAY 41-2272 that need the presence of a reduced haem group, the sGC activator BAY 58-2667 (and its chemical analogue BAY 60-2770) activate haem-free form of sGC [5], [10], [12], [13]. Oxidation of sGC haem to its ferric (Fe^3+^) form by ODQ causes a decrease in sGC protein levels [32] due to ubiquitination and proteasomal degradation [29]. The NO-independent sGC activator BAY 58-2667 binds with high affinity to the haem pocket of the enzyme and subsequently abrogates the effects of haem-oxidizing compounds on sGC protein levels [11], [32].

In the present study, prior incubation of platelets with ODQ did not affect the inhibition of platelet aggregation and [Ca^2+^]i levels by 8-Br-cGMP. However, ODQ significantly attenuated the inhibitory actions of SNP on platelet aggregation, adhesion, cGMP and [Ca^2+^]i levels, as expected. This NO donor produces anti-platelet activities through NO-, haem-dependent mechanisms [40], and in vascular smooth muscle it may present an additional mechanism involving endothelial NO production by cNOS activation [41]. In contrast to SNP, pre-incubation with ODQ rather potentiated the inhibitory effects of BAY 60-2770, as evidenced in the functional (aggregation and adhesion) and biochemical-molecular assays (cGMP generation, intracellular Ca^2+^ levels and integrin *α_IIb_β_3_* activation). Pre-incubation with ODQ also enhances the anti-aggregating effects and cGMP production of the sGC activator BAY 58-2667 in ADP-activated rat platelets [42]. Therefore, the higher production of cGMP in response to BAY 60-2770 in the presence of ODQ is likely to efficiently antagonize the increased intracellular Ca^2+^ levels in activated platelets, causing a further inhibition of the platelet function. BAY 60-2770 targets preferentially the sGC when the enzyme is under the haem-free form, and protection against oxidation-induced degradation seems to be a general feature of the haem-independent sGC activator BAY 58-2667 [11]. Moreover, the pathogenesis of cardiovascular diseases has been associated with inappropriate activation of sGC where the haem moiety is already found oxidized [32]. Accordingly, washed platelets isolated from coronary artery disease patients show increased levels of phosphorylated VASP compared with control group, which is further enhanced by ODQ [34].

Platelet activation is also inhibited by cAMP-elevating agents [43], [44] such as the stable prostacyclin analogue iloprost. The former sGC agonist YC-1 inhibits human neutrophil functions and accelerates resequestration of cytosolic Ca^2+^ through activation of the cAMP/protein kinase A pathway (PKA) [44], [45]. The sGC stimulator BAY 41-2272 has also been shown to present antiproliferative effects [46] through cAMP-dependent protein kinase PKA processes [47]. Moreover, BAY 41-2272 increases the cAMP levels in human eosinophils [48] and human myelomonocytic THP-1 cell line [49], despite to a much lesser extent than the cGMP levels and with little biological importance. However, in the present study, BAY 60-2770 failed to significantly elevate the cAMP levels in human washed platelets above baseline, excluding a role for this cyclic nucleotide in BAY 60-2770-induced responses. In the same experimental conditions, iloprost markedly elevated the cAMP levels, which were largely attenuated by prior treatment with the guanylyl cyclase inhibitor SQ-22536.

It has been suggested that increased levels of oxidative stress in pathophysiological conditions lead to oxidation or even loss of the sGC haem group, rendering the enzyme insensitive to NO [29], [32]. Reductions of sGC protein levels have been reported in aorta from spontaneously hypertensive rats [50], [51] and hypercholesterolemic rabbits [52]. ODQ has been shown to largely reduce the protein levels of α1 and β1 sGC subunits in both cGMP reporter cell line and porcine endothelial cells, which are prevented by the sGC activator BAY 58-2667 [11], [32]. BAY 58-2667 also increased the β1 (but not α1) subunit beyond control levels endothelial cells, as observed either in the presence or absence of ODQ [11], [32]. In our present study, we evaluated the effects of BAY 60-2770 in the protein levels of α1 and β1 sGC subunits under normal and haem-oxidizing conditions. Similarly to cGMP reporter cell line and porcine aortic endothelial cells, ODQ significantly reduced the protein levels of α1 and β1 sGC subunits in human platelets, which was restored to baseline by co-incubation with BAY 60-2770.

In summary, our present study shows that the sGC activator BAY 60–2770 presents anti-aggregating and anti-adhesive properties in human washed platelets due to cGMP generation, and subsequently reductions of intracellular Ca^2+^ levels and hence lower *α_IIb_β_3_* activation. Oxidation of the sGC haem with ODQ largely enhances the cGMP production causing further reductions in cytosolic Ca^2+^ and *α_IIb_β_3_* activation, with no involvement for the cAMP-dependent pathway. BAY 60-2770 also prevents the ODQ-induced decrease in protein levels of α1 and β1 sGC subunits. The marked anti-platelet activity of BAY 60-2770 under haem oxidation could be of therapeutic interest in cardiovascular diseases associated with atherothrombotic events.

## Methods

### Human washed platelet preparation

The study was approved by the Ethics Committee of State University of Campinas (UNICAMP; protocol number 487/2011). Written informed consent was obtained from every participant before blood donation. Human blood from healthy volunteers who had not received any medication within the previous 10 days was collected in ACD-C, (13 mM sodium citrate, 13 mM citric acid and 11 mM glucose, one volume of ACD-C for nine volumes of blood). The whole blood was centrifuged at room temperature (200 *g*, for 15 min) to obtain the platelet-rich plasma (PRP). Five milliliters of platelet-rich plasma (PRP) were added to 7 ml of washing buffer (140 mM NaCl, 0.5 mM KCl, 12 mM trisodium citrate, 10 mM glucose, 12.5 mM saccharose, pH 6.0), and again centrifuged at 800 *g* for 12 min. The platelets were gently suspended in Krebs solution containing (mM) 118 NaCl, 25 NaHCO_3_, 1.2 KH_2_PO_4_, 1.7 MgSO_4_, and 5.6 glucose (pH 7.4). The platelet number was adjusted to 1.2×10^8^ platelets/ml in the presence of 1 mM CaCl_2_.

### Experimental design

Platelet suspension (1.2×10^8^ platelets/ml) was pre-incubated for 3 min with the soluble guanylyl cyclase inhibitor ODQ (10 µM) or its correspondent vehicle DMSO (0.5% v/v). Next, platelets were incubated with BAY 60-2770 (0.001 to 10 µM), DMSO (0.5% v/v) or sodium nitroprusside (SNP, 3 µM; used as positive control) for another 3 min. Platelets were then stimulated with either collagen (2 µg/ml) or thrombin (0.1 U/ml) to perform the aggregation assays, cyclic nucleotide measurements (cAMP and cGMP), intracellular Ca^2+^ level measurements and integrin *α_IIb_β_3_* activation (see details below). The final concentration of DMSO never exceeded 1%, which does not interfere with functional or biochemical assays.

### Platelet aggregation

Platelet aggregation was performed with an optical aggregometer (Chrono-log, Kordia Life Sciences, Leiden) at 37°C with 400 µl of washed platelets placed in glass cuvettes containing a disposable stir bar for constant stirring. Platelet aggregation was carried out in collagen (2 µg/ml) and thrombin (0.1 U/ml)-stimulated platelets in the absence and in the presence of ODQ and/or BAY 60-2770, DMSO or SNP, as detailed above. The maximal aggregation (%) was calculated using the Aggrolink Software (Chrono-log). Krebs solution without vehicle (depending on the experimental protocols) provided a signal representing 0% aggregation.

### Platelet adhesion assay

Adhesion assay was carried out according to previous studies [53]. Briefly, 96-well microtiter plates were coated (overnight at 4°C) by adding 50 µl per well of human fibrinogen (50 µg/ml). The wells were washed twice with Krebs solution. The non-specific adhesion was blocked by incubation of wells with 1% BSA (1 h, 37°C), after which plate was carefully washed. Platelet suspension (50 µl) was added to each well (containing 6×10^6^ platelets). They were allowed to adhere to the wells for 30 min at 37°C. Thereafter, plates were carefully washed twice with Krebs solution to remove unattached platelets. Adherent platelets were quantified through the measurement of acid phosphatase activity. Wells containing adherent platelets were incubated with acid phosphatase substrate solution (0.1 M citrate buffer, pH 5.4, containing 5 mM *p*-nitrophenyl phosphate and 0.1% Triton X-100). After 1 h of incubation at room temperature, the reaction was stopped and the color was developed by the addition of NaOH. The *p*-nitrophenol produced in reaction was measured with a microplate reader at 405 nm (Synergy^TM^ H1 Hybrid Reader, Biotek, USA). The percentage of adherent cells was calculated on the basis of a standard curve obtained with known number of platelets (0.3 to 6×10^6^ platelets/well). All experiments were performed in triplicate.

### Extraction and measurement of cGMP and cAMP

Washed platelets (1.2×10^8^ platelets/ml) were pre-incubated with the phosphodiesterase inhibitor 3-isobutyl-l-methyl-xanthine (IBMX, 1 mM) for 20 min. Before addition of collagen (2 µg/ml) or thrombin (0.1 U/ml), platelet suspension (200 µl) was pre-incubated with ODQ and BAY 60-2770 (and respective control vehicle). Sodium nitroprusside (3 µM) or iloprost (0.3 µM) was used as a positive control for cGMP and cAMP, respectively. The reaction was interrupted by the addition of cold-acidified absolute ethanol (67%, v/v), and samples were vigorously agitated for 30 s. Cell samples were centrifuged (4.000 *g*, 30 min at 4°C). Supernatants were dried at 55–60°C under a stream of nitrogen. Preparation of tracer samples, standards and incubation with antibody were performed as described in commercially available kits (Cayman Chemical Cyclic GMP or AMP EIA kit, Ann Arbor, MI, USA). The assays were performed in duplicates. The limit of detection is 1 pmol/ml for cGMP and 0.1 pmol/ml for cAMP.

### Measurement of intracellular Ca^2+^ levels [Ca^2+^]_i_

Measurement of intracellular Ca^2+^ assay was carried out according to a previous study [54]. Briefly, the PRP obtained from ACD-C anti-coagulated blood were incubated with the fluorogenic calcium-binding dye FluoForte (10 µM) and pluronic 0.1% for 30 min at room temperature. Platelet suspension was then centrifuged at 800 *g* for 12 min. The pellet was gently resuspended in Krebs solution, and the number of platelets was adjusted to 1.2×10^8^ platelet/ml. Platelet aliquots of 300 µl were incubated with BAY 60-2770 (or DMSO) or SNP, in the absence and in the presence of ODQ (10 µM). The samples were analyzed using Fluorometer (Synergy^TM^ H1 Hybrid Reader, Biotek, USA). The external Ca^2+^ concentration was adjusted to 1 mM with CaCl_2_. Following equilibration for at least 3 min, thrombin (0.1 U/ml) or collagen (2 µg/ml) was added to platelet suspension. To verify the Ca^2+^ mobilization from internal storage sites alone, 10 µM EGTA was added to chelate the extracellular Ca^2+^. The fluorescence was monitored continuously with monochromator settings of 410 nm (excitation) and 514 nm (emission). The [Ca^2+^]_i_ levels was determined by applying the following equation [35]:


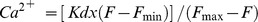


where *Kd*  = 389, *F is* fluorescence of the sample, *F_min_* is minimum fluorescence and *F_max_* is maximum fluorescence.

### Conformational changes in integrin *α_IIb_β_3_* (GPIIb/IIIa)

Conformational changes in integrin *α_IIb_β_3_* were assessed by binding to the FITC labeled PAC-1 (which recognizes only the conformationally activated GPIIb/IIIa) [55]. Platelet suspension (20 µL; 1.2×10^8^ platelets/ml) was pre-incubated with ODQ and BAY 60-2770 (0.01–10 µM) at room temperature for 3 min. Next, the platelets were incubated with either 10 µl of PAC-1 solution (FITCPAC-1; 25 µg/ml) or 10 µl of control antibody solution (FITC mouse IgM, same dilution of PAC-1 solution), after which collagen (2 µg/ml), thrombin (0.1 U/ml), or Krebs solution was added for 15 min. To minimize the presence of aggregates in the platelet samples, 450 µl of Krebs solution were added. Mean fluorescence was acquired in flow cytometer (FACSCalibur, BD, Franklin Lakes, NJ USA) equipped with a 488 nm wavelength argon laser using the FL1 channel. Platelets were identified by the forward and side scatter signals in a dot plot graphic in which a population was gated. Ten thousand events were acquired within the gate and their fluorescence was depicted in a histogram in log scale. Mean fluorescence was considered as a parameter to describe binding intensity to FITC labeled PAC-1.

### Western blotting

Platelets pellet were homogenized in a SDS lysis buffer with a sonicator (Fisher Scientiffic, Pittsburgh, PA, USA) for 5 sec and centrifuged (12,000× *g*, 4°C, 20 min) to remove insoluble material. Protein concentrations of the supernatants were determined by the Bradford assay, and equal amount of protein from each sample (50 μg) was treated with Laemmli buffer containing dithiothreitol 100 mM. Samples were heated in a boiling water bath for 5 min and resolved by SDS-PAGE. Electrotransfer of proteins to nitrocellulose membrane was performed for 60 min at 15 V (constant) in a semi-dry device (Bio-Rad, Hercules, CA, USA). Nonspecific protein binding to nitrocellulose was reduced by pre-incubating the membrane overnight at 4°C in blocking buffer (0.5% non-fat dried milk, 10 mMTris, 100 mMNaCl, and 0.02% Tween 20). Detection using specific antibodies, HRP-conjugated secondary antibodies, and luminal, p-Coumaric acid and H_2_O_2_. One min after incubation, x-ray sensitive films were exposed to the membranes and marked by a chemiluminescent signal. The individual sGC subunits were detected by using polyclonal antibodies directed against specific epitopes of the α_1_ subunit (AbCam Technology, Cambridge, England, UK), β_1_ subunit (NovusBiologicals, Littleton, CO, USA) and anti GAPDH was from Santa Cruz Biotechnologie (Santa Cruz, CA, USA). Protein levels were normalized with GAPDH. Densitometry was performed using the Scion Image software (Scion Corporation, Frederick, MD), and results represented as the percentage in relation to control protein expression.

### Drugs and materials

Bovine serum albumim (BSA, from bovine serum), fibrinogen (fraction I from human plasma), phosphatase substrate (p-Nitrophenyl phosphate, sodium), thrombin (from human plasma), 3-isobutyl-1-methylxanthine (IBMX), 1H-[1], [2], [4]oxadiazolo[4,3-a]quinoxalin-1-one (ODQ), 9-(tetrahydro-2-furanyl)-9H-purin-6-amine (SQ 22-536), pluronic P-123, dimethyl sulfoxide (DMSO), sodium nitroprusside (SNP) were purchased from Sigma Chem. Co. (St. Louis, MO, USA). Iloprost and Fluoforte^TM^ reagent were obtained from Schering AG (Berlin, Wedding, Germany) and Enzo Life Sciences International (Butler Pike, PA, USA), respectively. BAY 60-2770 (acid 4-({(4-carboxybutyl) [2-(5-fluoro-2-{[4-(trifluoromethyl) biphenyl-4-yl] methoxy} phenyl) ethyl] amino} methyl) benzoic) was obtained from Pharma Research Center, Bayer AG (Wuppertal, Germany). Collagen was purchased from Chrono-log Corporation (PA, USA). Cyclic AMP and cyclic GMP enzyme immunoassay (EIA) kits were purchased from Cayman Co. (Ann Arbor, MI, USA). PAC-1 FITC monoclonal mouse anti-platelet *α_IIb_β_3_* antibodies were obtained from Becton Dickinson (San José, CA, USA).

### Statistical analysis

Data are shown as mean ± SEM and n indicates the number of human subjects. One-way analysis of variance (ANOVA) followed by Newman–Keuls or Dunnett multiple comparison test was performed. Students t two-tailed test for paired or unpaired experiments was used when appropriate. Differences were considered to be statistically significant when p<0.05.
